# Competing therapy options for severe amlodipine poisoning—lessons learned: a case report

**DOI:** 10.1186/s13256-025-05781-3

**Published:** 2026-01-09

**Authors:** Yvonne Kuhn, Julia Fischbach, Christian Putensen, Stefan Felix Ehrentraut

**Affiliations:** Anaesthesiology, Intensive Care and Emergency Medicine, Clinic for Anaesthesiology and Intensive Care, University Hospital Bonn, Venusberg-Campus 1, 53127 Bonn, Nordrhein-Westfalen Germany

**Keywords:** Amlodipine intoxication, Extracorporeal membrane oxygenation, Vasoplegia, Hydroxycobalamin, Plasma exchange

## Abstract

**Background:**

We report two cases of severe amlodipine poisoning (> 1000 mg) during a suicide attempt.

**Case presentation:**

Both patients (male, white, aged 70 and 28 years, respectively) experienced severe vasoplegic shock and were referred to us from regional hospitals via our extracorporeal membrane oxygenation hotline. In addition to the recommended therapy, both patients underwent albumin dialysis. Despite this, the vasopressor demand remained sky high, in both cases above 2 µg/kg/minute norepinephrine. Ultimately, both patients required veno-arterial extracorporeal membrane oxygenation support. There were slight differences in treatment; one patient received lipid emulsion and the other hydroxycobalamin, both of which caused interesting technical difficulties with continuous renal replacement therapy. Finally, we were able to wean both patients off extracorporeal support as well as all vasopressors. Both were discharged from the intensive care unit.

**Conclusion:**

In cases so severe, swift action is of the essence and some of the available treatments cancel each other out and should be timed accordingly. Therefore, on the basis of our experiences and the standing recommendations we developed a treatment algorithm that takes not only the case severity but also the time frame in which the treatment should be administered into account.

## Background

Calcium channel blockers are common medications that play an increasing role in substance exposure and suicide. In the 2021 annual report of the American Association of Poison Control Centers, calcium channel blockers ranked 6th in the top 25 of fatal drug exposures [1].

Unfortunately, there is no antidote available, making treatment challenging. Among the various treatment regimens, there are no practice guidelines based on randomized controlled trials. Recently, a systemic review of existing case reports was published, assessing treatment methods in regard to hemodynamic parameters and mortality [2]. This is the second of two existing systemic reviews, the first one dating back to 2014 [3].

We report two cases of severe amlodipine poisoning. Both patients ingested over 1000 mg of amlodipine. In the systemic review by Baid *et al*., 18 case reports were included, most of which ingested 500 mg of amlodipine or less. The outcomes in patients with higher overdoses were mixed (600 mg of amlodipine: patient was discharged from the intensive care unit [ICU], 800 mg of amlodipine: patient expired, 840 mg of nimedipine: patient sustained hypoxic brain injury); none had been reported with an overdose of over 1000 mg of amlodipine [4–6]. Indeed, the German poison control center has deemed this dose lethal.

Therefore, we want to report on our two cases regarding this high overdose and the new therapy recommendations published.

## Case presentation

### Patient 1

We describe the case of a male, white, 70-year-old patient who ingested 1000 mg of amlodipine in a suicide attempt, with no prior psychiatric history.

**Fig. 1:**
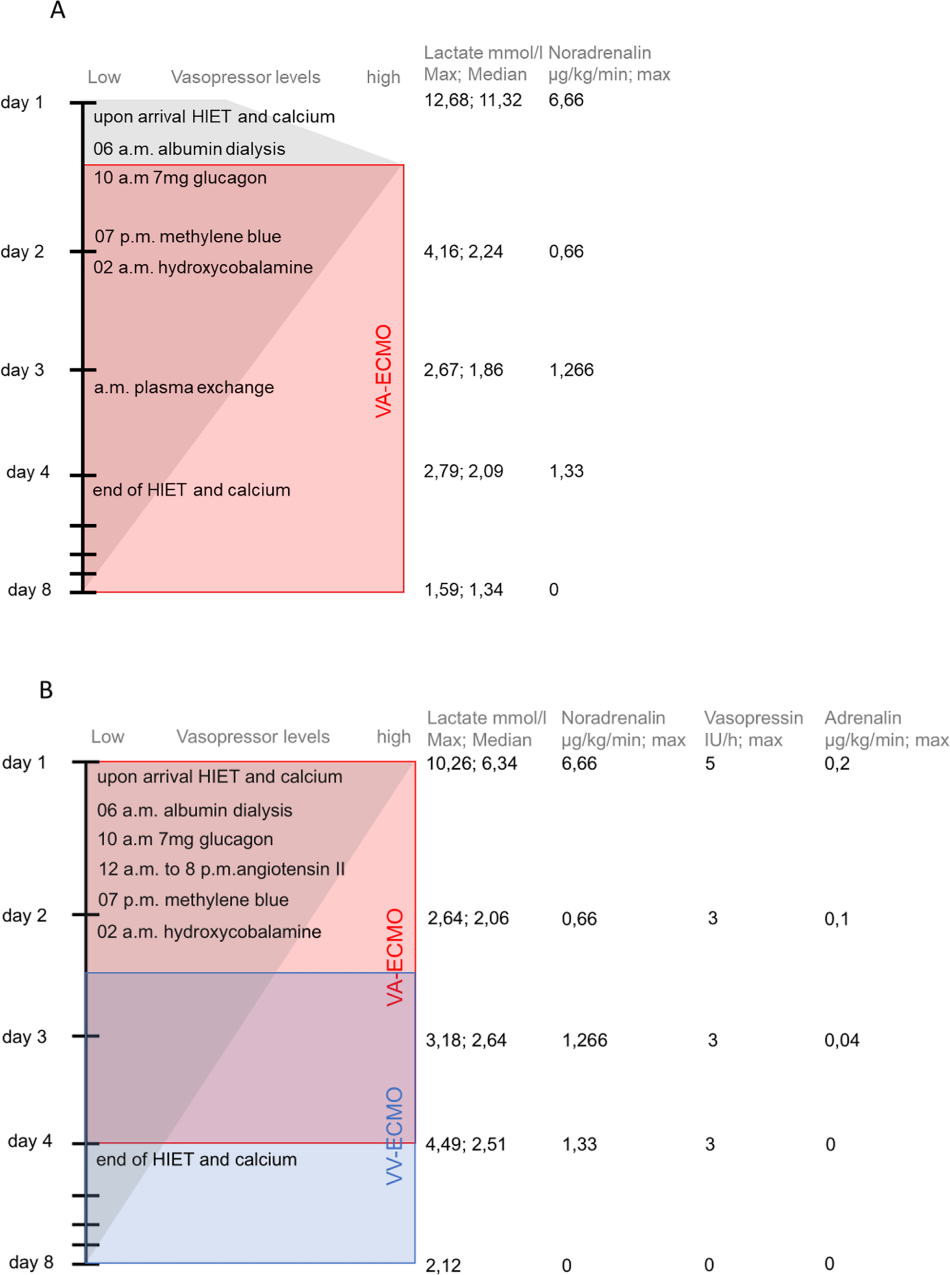
Timelines for patients 1 (**A**) and 2 (**B**)

He was referred to us for extracorporeal membrane oxygenation (ECMO) evaluation after an external hospital managed him with over 3 µg/kg/minute of noradrenaline to maintain a mean arterial pressure (MAP) of 60 mmHg (maximum daily vasopressor values are listed within Fig. 1A). Treatment included intravenous normal saline, calcium gluconate, high-insulin euglycemia therapy, as well as 20% intravenous intralipid emulsion, as recommended by the poison control center [7]. Despite these measures, the patient’s condition did not improve, and he was transferred to our ICU. Upon arrival, his vasopressor requirement decreased to 2 µg/kg/minute of noradrenaline, allowing transport without veno-arterial (VA)-ECMO.

In our ICU, the patient was awake (Glasgow Coma Scale [GCS] 15) and breathed spontaneously. We administered glucagon, starting with a 10 mg bolus followed by a 5 mg bolus to a total of 15 mg, as well as methylene blue. The patient’s hemodynamic status did not improve. We then started albumin dialysis [8]. With this we faced some interesting technical issues. Even though we used a high-flux filter with a sieving coefficient of over 0.9 for triglycerides and LDL, a secondary membrane was formed by the intralipid emulsion that had been administered not hours prior. This caused a high transmembrane pressure and almost instantaneous filter clotting. Despite these difficulties, we were able to exchange 3 L of plasma. However, if a patient is already dependent on continuous hemodialysis, the use of an intralipid emulsion may be disadvantageous.

Unfortunately, the initial albumin dialysis had no noticeable positive impact on the overall situation; vasopressor demand remained high at approximately 2 µg/kg/minute noradrenaline. Furthermore, the patient began to develop combined respiratory-metabolic acidosis, respiratory failure, and encephalopathy requiring intubation and initiation of VA-ECMO. Under ECMO treatment the vasopressor demand declined significantly. Three days later, we performed another plasma exchange with fresh frozen plasma because of chronic liver failure. From then on, we were able to see a rapid decline in amlodipine blood levels, falling from above 140 ng/ml to 40 ng/ml (for reference, blood levels of 15 ng/ml are therapeutic) [9]. The ECMO was explanted after 8 days. Finally, the patient was discharged from the ICU and transferred back to the referring hospital with no lasting organ damage.

### Patient 2

The second case is that of a male, white, 28-year-old patient who ingested 1290 mg of amlodipine in a suicide attempt. He had a brief medical history of depression with suicidal thoughts, but so far, no suicide attempts.

At the referring hospital, he received activated charcoal and high-insulin euglycemia therapy but developed pulmonary dysfunction requiring intubation. He experienced progressive vasoplegia. His condition worsened, prompting an evaluation by our ECMO team.

At that point, the patient was in refractory vasoplegic shock. A maximal dosage of 6.6 µg/kg/minute noradrenaline, 4U/hour vasopressin, and 0.16 µg/kg/minute adrenaline were needed to maintain a mean arterial pressure of 65 mmHg (maximum daily vasopressor values are listed within Fig. 1B). A peripheral VA-ECMO was established onsite. We chose not to use lipid rescue owing to complications witnessed in Patient 1 and began albumin dialysis upon ICU admission.

Methylene blue was administered but showed little result. We decided to extend the regimen to angiotensin II acetate, reducing the noradrenaline dosage to around 2 µg/kg/minute. Glucagon was administered, starting with a bolus of 7 mg, followed by 7 mg/hour, the administration was stopped after only 1 hour owing to availability. In a recent case report, good results were achieved by off-label use of hydroxycobalamin [10]. On the basis of that we also administered hydroxycobalamin, which showed good results. The vasopressor dosages decreased to 0.25 µg/kg/minute noradrenaline.

However, hydroxycobalamin caused chromaturia. This discoloration did not only occur in urine, but also in the dialysis filtrate, which triggered machine safety protocols, causing it to stop treatment. The safety protocols had judged the red discoloration to be hemolysis and we were unable to circumvent this and restart dialysis. The discoloration lasted approximately 72 hours. Fortunately, Patient 2 was not solely dependent on continuous dialysis, and the break did not cause any complications.

By day 3, the hemodynamic condition improved, allowing explantation of the arterial ECMO cannula. The patient developed acute respiratory distress syndrome and remained on veno-venous (VV)-ECMO for another 10 days. The patient recovered without lasting organ damage and was transferred to our psychiatric ward with good neurological outcome for further treatment.

## Discussion and conclusion

Severe amlodipine poisoning causes prolonged vasoplegic shock. This can be managed using proper treatment and quick action until plasma levels decrease. Several treatment options exist for amlodipine poisoning, including well-established ones, such as high-insulin euglycemia therapy, and less common ones, such as albumin dialysis, methylene blue, and hydroxocobalamin.

High-insulin euglycemia therapy is the standard and improves survival [2, 7, 11].

Glucagon is also a long standing recommendation but was limited in availability in our case [12]. Therefore, it had little effect on hemodynamic parameters, as opposed to cases where glucagon could be administered for longer periods [13]. Since amlodipine intoxications are on the rise, we advise evaluating the availability at your center [1, 7].

Albumin dialysis was first described for calcium channel blocker removal in 2006 [14]. Other than conventional hemodialysis, it can help reduce the blood levels of albumin-bound amlodipine. Consequently it improves survival [2]. However, the process is time consuming, as multiple dialyses, over the course of several days, are needed to remove amlodipine (depending on the initial amount ingested) from the blood stream. Hence, it is a supportive therapy with only indirect causal effects.

Intralipid emulsion can be used to improve hemodynamic parameters in a wide range of substance exposures, among them, calcium channel blockers [15, 16]. Even though it is associated with better survival and positive effects on hemodynamics, in Patient 2 we deliberately decided against administering it to not compromise albumin dialysis or the integrity of the ECMO membrane [2]. There have been several reports in the past about hypertriglyceridemia causing clogging of dialysis filters [17]. This was also the case in Patient 1, where triglyceride levels reached close to 7000 mg/dl following lipid rescue.

Methylene blue, a nitric oxide scavenger, has some positive effects on hemodynamics, but lacks sufficient evidence for standard use and showed poor results in our case [2, 18]. We therefore cannot recommend it.

Hydroxycobalamin as a counteracting agent for vasoplegic shock was first described in a patient post-thoracic surgery [19]. Since then, several reviews have been published on its use in perioperative vasoplegia. However, the fact that hydroxycobalamin causes chromaturia, disrupting dialysis and plasma exchange has to be taken into account [20]. For calcium channel blocker intoxication there has only been one case report so far, which reported a swift response and recovery [10]. While we also witnessed a significant decrease in vasopressors, the time frame for administration should be chosen correctly.

Lastly, extracorporeal life support is recommended as a last resort for vasoplegic shock and has shown to be associated with an improved survival, left ventricular function, and blood pressure [2, 7].

**Fig. 2:**
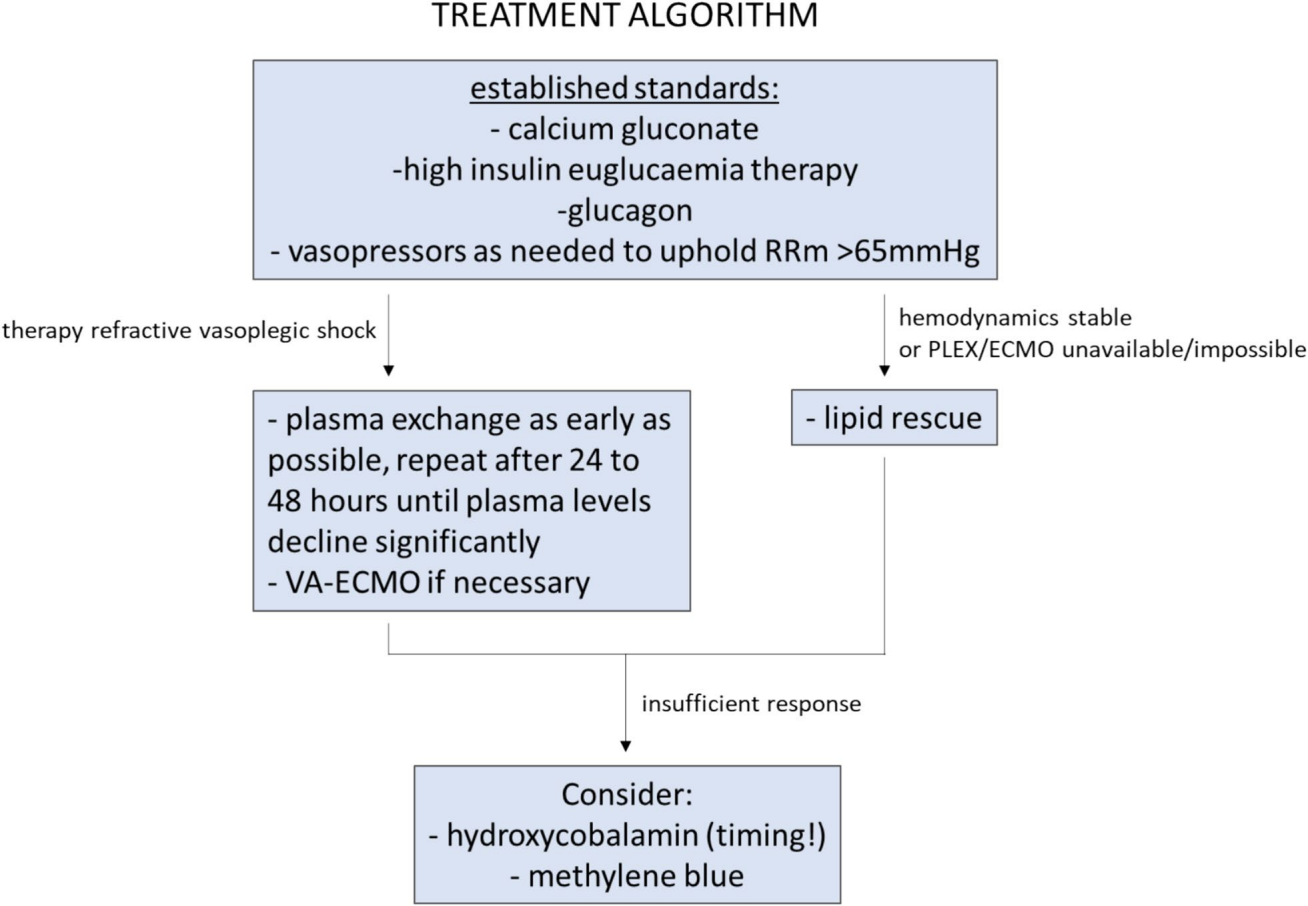
In-house treatment algorithm for amlodipine intoxication

Calcium antagonists are frequently used in suicide attempts [1]. Limited data is available, potentially because most cases are fatal and remain undetected prior to the initiation of treatment. In cases of severe amlodipine poisoning prolonged vasoplegic shock is inevitable. In our case, the combination of treatments and ECMO led to the patients’ full recovery. On the basis of these recommendations we established our own institutional algorithm for treating amlodipine intoxication, as shown in Fig. 2.

## Limitations

As with all case reports or small case series, several limitations must be acknowledged:

- The presented data are derived from only two cases, which inherently limits the extent to which the findings can be extrapolated.
- Given the use of multiple concomitant interventions (albumin dialysis, insulin/glucagon therapy, methylene blue, and hydroxocobalamin), it is not possible to attribute specific effects to any single treatment.
- The proposed treatment algorithm reflects our institutional experience and should be interpreted as such; its generalizability to other settings has not been established.

## Data Availability

The data that support the case report are available upon request. The data are restricted owing to anonymization and will be shared with qualified researchers for specified purposes. To request access to the data, please contact the corresponding author.

## Abbreviations

ECMO: Extracorporeal membrane oxygenation
VV-ECMO: Veno-venous extracorporeal membrane oxygenation
VA-ECMO: Veno-arterial extracorporeal membrane oxygenation
CRRT: Continuous renal replacement therapy
MAP: Mean arterial pressure
ICU: Intensive care unit
GCS: Glasgow Coma Scale
µg: Microgram
kg: Kilogram
U: Units
ng: Nanogram
ml: Milliliter
mg: Milligram
dl: Deciliter
LDL: Low-density lipoprotein
